# Risk factors for suicidal thoughts in informal caregivers: results from the population-based Netherlands mental health survey and incidence Study-2 (NEMESIS-2)

**DOI:** 10.1186/s12888-019-2317-y

**Published:** 2019-10-28

**Authors:** Karlijn J. Joling, Margreet ten Have, Ron de Graaf, Siobhan T. O’Dwyer

**Affiliations:** 1 Department of General Practice and Elderly Care Medicine, Amsterdam UMC, Vrije Universiteit Amsterdam, Amsterdam Public Health Research Institute, de Boelelaan, 1117 Amsterdam, The Netherlands; 2 Netherlands Institute of Mental Health and Addiction, Da Costakade 45, 3521 VS Utrecht, The Netherlands; 3 University of Exeter Medical School, South Cloisters, St Luke’s Campus, Exeter, EX1 2LU UK

**Keywords:** Informal caregivers, Suicidal ideation, Suicidal thoughts, Risk factors, Population study

## Abstract

**Background:**

Previous research suggests that family caregivers contemplate suicide at a higher rate than the general population. Much of this research has been disease specific and in relatively small samples. This study aimed to compare suicidal thoughts between non-caregivers and informal caregivers of people with a variety of conditions, in a large representative sample, and to identify significant risk factors.

**Methods:**

The general population study NEMESIS-2 (N at baseline = 6646) included 1582 adult caregivers at the second wave (2010–2012) who also participated at the third wave (2013–2015). Suicidal thoughts were assessed over 4 years, with the Suicidality Module of the Composite International Diagnostic Interview 3.0. The presence of suicidal thoughts was estimated and risk factors for suicidal thoughts were assessed with logistic regression analyses adjusted for age and gender.

**Results:**

Thirty-six informal caregivers (2.9%) reported suicidal thoughts during the 4 year study period. The difference between caregivers and non-caregivers (3.0%) was not significant. Among caregivers, significant risk factors for suicidal thoughts included being unemployed, living without a partner, having lower levels of social support, having a chronic physical disorder, a mood disorder or an anxiety disorder, and having impaired social, physical and emotional functioning. These risk factors were also found in non-caregivers. No caregiving-related characteristics were associated with suicidal thoughts.

**Conclusion:**

There was no elevated rate of suicidal thoughts in caregivers and risk factors for suicidal thoughts in caregivers were consistent with risk factors in non-caregivers. No association between caregiving characteristics and suicidal thoughts was found. Caregivers with limited resources and in poorer health might still benefit from prevention and intervention efforts.

## Introduction

Family caregivers provide the cornerstone of care for people living with long-term illnesses and disabilities, and in Europe alone there are more than 100 million of them [1]. They provide essential support across a range of domains, including physical health, mental health, household management, financial management, and legal administration [2]. As well as making a significant contribution to the wellbeing of individuals, family caregivers also make a social and economic contribution, saving governments a substantial amount of money in formal care [3].

More than 50 years’ worth of research, however, has shown that caring takes a significant toll on the physical health, mental health, social engagement, career prospects, and financial security of family caregivers [4]. More recently, research has shown that specific groups of family caregivers may also be a high risk group for suicide. For example, studies of people with dementia [5–7], HIV [8, 9], and cancer [10, 11] have all identified suicidal thoughts in family caregivers, with some reporting rates of suicidal thoughts more than four times that of the general population [7]. This research has also been global in its focus, including studies of caregivers in Australia [7, 12], the UK [13, 14], the Netherlands [5], the USA [8, 10, 15], Asia [6, 11, 16], and Africa [9, 17].

The vast majority of this research, however, has used purposive, and relatively small, study samples. And, of the few studies that have used large representative samples [12, 14, 16], only two have compared caregivers with non-caregivers. In a study of female caregivers in Australia, O’Dwyer and colleagues (2014) found that caregivers had significantly higher rates of suicidal thoughts than non-caregivers (7.1 versus 5.7%). In a mixed-gender study in the UK, Stansfeld and colleagues (2014) found that caregivers were twice as likely to report thinking about suicide in the last week as non-caregivers. Unfortunately, both studies lacked information about characteristics of caregiving, such as the disease of the person being cared for and the relationship with the care recipient. Rates of suicidal thoughts in family caregivers may vary according to the illness or disability for which they are providing care, and the nature of their relationship with the care receiver [16]. The lack of a representative sample and a comparison with non-caregivers in the majority of the current research on suicidal thoughts in family caregivers significantly limits the conclusions that can be drawn. More high quality research is required to understand how the rate of suicidal thoughts in caregivers compares with non-caregivers.

The aim of this study was to compare the rate of suicidal thoughts in caregivers and non-caregivers in a large, representative sample, and to identify significant risk factors for suicidal thoughts in caregivers that might provide directions for prevention and intervention.

## Methods

### Study design and source population

NEMESIS-2 (Netherlands Mental Health Survey and Incidence Study-2) is a psychiatric epidemiological cohort study in the Dutch general population aged 18 to 64 years at baseline. It is based on a multi-stage, stratified random sampling procedure of households, with one respondent randomly selected in each household. The study was approved by the Medical Ethics Review Committee for Institutions on Mental Health Care. Participants provided written informed consent at each wave. The provision of informal care was assessed at the second wave (T1), and we therefore used the data of T1 and the subsequent wave (T2) for the current study to investigate suicidal thoughts.

In the first wave of NEMESIS-2 (T0; November 2007 to July 2009), 6646 respondents were interviewed (response rate: 65.1%; average duration: 95 min). This sample was nationally representative, although younger subjects were somewhat underrepresented [18]. All T0 respondents were approached for first follow-up (T1; November 2010 to June 2012), 3 years after T0. A total of 5303 people were available for interview (response rate: 80.4%, with those deceased excluded; duration: 84 min). All T1 respondents were approached for second follow-up (T2; November 2013 to June 2015), 3 years after T1. A total of 4618 people were interviewed again (response rate 87.8%; duration: 83 min). Attrition between T0 and T2 was significantly associated with 12 month bipolar disorder at T0 after controlling for sociodemographic variables, but not with any other mental disorders [19].

### Data collection and procedures

The face-to face interviews were laptop computer-assisted and mainly conducted at the respondent’s home. The interviews were conducted by trained professional interviewers who were selected for their experience with systematic face-to-face data collection, experience with sensitive topics, and ability to achieve a good response rate in other studies.

Fieldwork was monitored over the entire data collection period by the NEMESIS-investigators and the fieldwork agency. For more information on quality checks of the data and a more detailed description of the design and fieldwork, see [18].

### Informal caregiving

At the second wave (T1), respondents were asked whether they provided unpaid care in the previous 12 months, to a family member, partner or friend who needed care because of physical problems, mental problems, or ageing (*n* = 1759). Of these caregivers, 1582 (89.9%) also participated in the third wave (T2) and were included in the current study. This enabled us to investigate the presence of suicidal thoughts over a period of 4 years (1 year before T1, and in the 3 years between T1 and T2).

### Presence of suicidal thoughts

Suicidal thoughts were assessed with the Suicidality Module of the Composite International Diagnostic Interview (CIDI) version 3.0, a fully structured lay-administered diagnostic interview for common mental disorders. This instrument was developed for use in the World Mental Health Survey Initiative [20].

At T1, respondents were asked about thoughts of suicide in the previous 12 months (“Have you seriously thought about committing suicide?”) and at T2 they were asked about thoughts of suicide in the previous 3 years (i.e. since T1). At both waves, to encourage people to report their thoughts, plans or attempts of suicide, the experiences were not mentioned but listed in a booklet and referred to by number (“event A”), as was done in other studies as well (e.g. [21]). For the purposes of this analysis, the presence of suicidal thoughts was defined as having suicidal thoughts at any point during 4 years (i.e. from the 12 months preceding T1 until T2).

### Risk factors for suicidal thoughts

A broad set of variables was used to identify potential risk factors for suicidal thoughts. These were all assessed at T1, except for educational level, which was assessed at T0. Obviously, the caregiving-related characteristics were not assessed in non-caregivers.

- *Sociodemographics* included: age; gender; educational level (primary, basic vocational or lower secondary education, higher education); employment status (in paid employment, not in paid employment), living with a partner (yes, no); and, parental status (cohabiting with child(ren) yes/no).
- *Caregiving-related characteristics* included: the type of relationship with the care recipient (partner, first-degree family [e.g. parents, siblings or children], other family [e.g. in-laws] or friend); number of care recipients (one, more than one); cohabiting with the care recipient (yes, no); reasons for care (physical problems, mental problems, ageing, dementia); type of care (domestic chores, personal care/nursing, emotional support/supervision, practical care); time spent giving care (0–7 h per week, 8 or more hours per week); and, duration of care (less than 1 year, 1 year or more).
- *Other characteristics* included:
  - ◦ *Perceived social support* from three sources (partner, family or friends, neighbours) was measured with two questions on instrumental and emotional support from each source. These referred to the extent respondents could rely on them for help if they had a problem and could open up to them if they needed to talk about worries. The four response categories ranged from one (not at all) to four (a lot). Perceived social support was calculated as the mean score from at least two sources—because not all respondents had a partner at the time of interview.
  - ◦ *Chronic physical disorders* were identified with a standard checklist of 17 chronic physical disorders, treated or monitored by a medical doctor in the 12 months prior to T1, including: asthma; chronic obstructive pulmonary disease; chronic bronchitis; emphysema; severe heart disease; heart attack; hypertension; stroke; stomach or intestinal ulcers; severe intestinal disorders; diabetes; thyroid disorder; chronic back pain; arthritis; migraine; impaired vision or hearing; or, any other chronic physical disorder [22].
  - ◦ *Mood disorders* (major depression, dysthymia and bipolar disorder) and *Anxiety disorders* (panic disorder, agoraphobia, social phobia, specific phobia and generalized anxiety disorder) in the last 12 months were identified with the CIDI 3.0 [23]. Clinical calibration studies in various countries found that the CIDI 3.0 assesses common mental disorders with generally good validity in comparison with blinded clinical reappraisal interviews [20].
  - ◦ The presence of ten *negative life events* (e.g. death of a relative or friend, divorce) in the 12 months prior to T1 were measured, based on the Brugha Life Events scale [24].
  - ◦ *Role functioning* was assessed in the past 4 weeks for the *physical, mental and social domains* from the Medical Outcomes Study Short Form Health Survey (SF-36) [25]. All three functioning scales ranged from 0 (low functioning/ ill health) to 100 (high functioning), and were dichotomized into high functioning (score 100) versus impaired functioning (score < 100).

### Statistical analysis

First, we compared the baseline characteristics of caregivers who dropped out at T2 with caregivers who completed T2 by performing logistic regression analysis. Second, the rate of suicidal thoughts was calculated as the number of caregivers who reported these thoughts at any point during the 4 years, i.e. between the 12 months prior to T1 and T2. We checked whether the rate of suicidal thoughts differed between informal caregivers and non-caregivers, with a Chi-squared test using weighted data to correct for differences in the response rates in several sociodemographic groups at both waves and differences in the probability of selection of respondents within households at baseline [18]. Logistic regression analysis (unweighted), adjusted for age and gender, was used to identify factors that were significantly associated with suicidal thoughts, in both the informal caregivers and non-caregivers. Because of the low numbers of caregivers with suicidal thoughts we did not perform multivariate analyses with all potential risk factors. Statistical significance was considered as two-tailed *p* < 0.05. Analyses were conducted in SPSS 22 and STATA 12.1.

## Results

### Sample characteristics

The characteristics of the study sample are described in Table 1. Informal caregivers had an average age of 50 years old and 65% was female. The majority (75%) were living with a partner, and 43% cohabited with children. More than half (57%) were caring for a first degree family member, and the vast majority (83%) lived separately from the care recipient. About one-third provided eight or more hours of care per week, and 49% had provided care for longer than 1 year. The main reasons for care were physical (59%) and mental (30%) problems.

**Table 1:** Sample characteristics and risk factors for suicidal thoughts in informal caregivers, adjusted for age and gender

	Total *n = 1582*	Suicidal thoughts *n = 36*	No suicidal thoughts *n = 1546*	Model adjusted for age and gender		
	n (%)	n (%)	n (%)	OR	95% CI	p
*Sociodemographics*						
Age, mean (SD)	50.2 (11.5)	49.2 (10.0)	50.2 (11.5)	0.99	0.97; 1.02	0.61
Female gender	1022 (64.6)	25 (69.4)	997 (64.5)	1.24	0.60; 2.54	0.56
Lower educational level^a^	469 (29.6)	15 (41.7)	454 (29.4)	1.79	0.90; 3.57	0.10
No paid employment	501 (31.7)	17 (47.2)	484 (31.3)	2.34	1.14; 4.80	0.02
Living without a partner	400 (25.3)	16 (44.4)	384 (24.8)	2.39	1.22; 4.66	0.01
Cohabitation with child(ren)	688 (43.5)	16 (44.4)	672 (43.5)	0.96	0.47; 1.95	0.91
*Caregiving characteristics*						
Relationship with care recipient						
Partner	191 (12.1)	6 (16.7)	185 (12.0)	1.54	0.63; 3.77	0.35
First-degree family	903 (57.1)	20 (55.6)	883 (57.1)	0.93	0.48; 1.82	0.84
Other family	453 (28.6)	10 (27.8)	443 (28.7)	0.94	0.45; 1.98	0.88
Friend	255 (16.1)	4 (11.1)	251 (16.2)	0.63	0.22; 1.79	0.38
More than one care recipient	615 (38.9)	17 (47.2)	598 (38.7)	1.41	0.72; 2.73	0.32
Living with care recipient	266 (16.8)	9 (25.0)	257 (16.6)	1.69	0.79; 3.64	0.18
Providing care for 8 or more hours per week	507 (32.0)	11 (30.6)	496 (32.1)	0.93	0.45; 1.92	0.84
Providing care for longer than one year	777 (49.1)	21 (58.3)	756 (48.9)	1.52	0.77; 3.01	0.23
Reason for care						
Physical problems	938 (59.3)	21 (58.3)	917 (59.3)	0.96	0.49; 1.87	0.90
Mental problems	472 (29.8)	16 (44.4)	456 (29.5)	1.88	0.96; 3.66	0.07
Ageing	511 (32.3)	8 (22.2)	503 (32.5)	0.61	0.27; 1.35	0.22
Dementia	212 (13.4)	5 (13.9)	207 (13.4)	1.06	0.41; 2.79	0.90
Type of care						
Domestic work	915 (57.8)	22 (61.1)	893 (57.8)	1.12	0.56; 2.21	0.75
Personal care or nursing	419 (26.5)	13 (36.1)	406 (26.3)	1.56	0.78; 3.12	0.21
Emotional support	1419 (89.7)	34 (94.4)	1385 (89.6)	1.97	0.47; 8.30	0.36
Practical care	1232 (77.9)	28 (77.8)	1204 (77.9)	1.02	0.46; 2.27	0.96
*Other characteristics*						
Social support total score, mean (SD)	3.4 (0.6)	3.1 (0.8)	3.4 (0.59)	0.50	0.31; 0.80	< 0.01
Chronic physical disorder in last 12 months	753 (47.6)	27 (75.0)	726 (47.0)	3.75	1.72; 8.17	< 0.01
Mood disorder in last 12 months	72 (4.6)	12 (33.3)	60 (3.9)	12.32	5.80; 26.14	< 0.001
Anxiety disorder in last 12 months	100 (6.3)	9 (25.0)	91 (5.9)	5.23	2.35; 11.65	< 0.001
Negative life event in last 12 months	878 (55.5)	24 (66.7)	854 (55.2)	1.59	0.79; 3.21	0.19
Impaired social role functioning (< 100)	601 (38.0)	26 (72.2)	575 (37.2)	4.34	2.07; 9.10	< 0.001
Impaired physical role functioning (< 100)	215 (13.6)	15 (41.7)	400 (25.9)	4.73	2.39; 9.36	< 0.001
Impaired emotional role functioning (< 100)	420 (26.5)	20 (55.6)	200 (12.9)	3.58	1.83; 7.01	< 0.001

^a^primary basic vocational /lower secondary versus Higher secondary/ Higher professional/ university

Over the 3 year follow-up, 10% of the caregivers (*n* = 177) were lost to follow-up. None of the baseline variables were significantly associated with loss to follow-up.

### Suicidal thoughts

Across the 4 years, 36 of the caregivers (2.9% weighted percentage) reported suicidal thoughts (15 caregivers at T1, 21 caregivers at T2, and 6 caregivers at both measurements). The weighted prevalence of suicidal thoughts in informal caregivers did not differ significantly from people who did not provide informal care at T1 (2.9% versus 3.0%, weighted design based F(1,89), *p* = 0.89)).

### Risk factors for suicidal thoughts

Nine risk factors were significantly associated with suicidal thoughts in caregivers (Table 1): employment status, partner status, social support, physical health, mood disorder, anxiety disorder, social functioning, physical functioning and emotional functioning. Caregivers without paid employment were more than twice as likely to contemplate suicide than caregivers in paid employment, and caregivers living without a partner were more than twice as likely to contemplate suicide than those living with a partner. Caregivers with a chronic physical disorder were more than three times as likely to contemplate suicide than those without, while caregivers with a mood disorder were more than 12 times more likely to contemplate suicide than caregivers without. Caregivers with an anxiety disorder were more than 5 times more likely to contemplate suicide than those without. Caregivers with impaired social, physical and emotional role functioning were between 3 and 5 times more likely to contemplate suicide than those without impaired role functioning (see Table 1). Caregivers with higher levels of social support were less likely to report suicidal thoughts than those with lower levels of social support. No caregiving characteristics were significantly associated with suicidal thoughts, although if the reason for care was mental problems, this approached the level of statistical significance (OR 1.88, *p* = 0.07). In non-caregivers, the same risk factors were found as in informal caregivers (Table 2). In addition to these risk factors, a lower educational level and the experience of a negative life event in the last 12 months were also significantly associated with suicidal thoughts in non-caregivers.

**Table 2:** Sample characteristics and risk factors for suicidal thoughts in non-caregivers, adjusted for age and gender

	Total *n = 3036*	Suicidal thoughts *n = 86*	No suicidal thoughts *n = 2950*	Model adjusted for age and gender		
	n (%)	n (%)	n (%)	OR	95% CI	p
*Sociodemographics*						
Age, mean (SD)	46.7 (12.6)	46.1 (11.0)	46.7 (12.6)	1.00	0.98; 1.01	0.70
Female gender	1537 (50.6)	43 (50.0)	1494 (50.6)	0.97	0.63; 1.49	0.90
Lower educational level^a^	910 (30.0)	38 (44.2)	872 (29.6)	2.04	1.30; 3.20	< 0.01
No paid employment	741 (24.4)	35 (40.7)	706 (23.9)	2.76	1.70; 4.48	< 0.001
Living without a partner	907 (29.9)	43 (50.0)	864 (29.3)	2.41	1.57; 3.71	< 0.001
Cohabitation with child(ren)	1367 (45.0)	37 (43.0)	1330 (45.1)	0.90	0.57; 1.40	0.63
*Other characteristics*						
Social support total score, mean (SD)	3.4 (0.6)	2.9 (0.9)	3.4 (0.6)	0.33	0.25; 0.44	< 0.001
Chronic physical disorder in last 12 months	1200 (39.5)	53 (61.6)	1147 (38.9)	2.93	1.84; 4.67	< 0.001
Mood disorder in last 12 months	152 (5.0)	36 (41.9)	116 (3.9)	18.32	11.39; 29.46	< 0.001
Anxiety disorder in last 12 months	164 (5.4)	17 (19.8)	147 (5.0)	4.78	2.72; 8.38	< 0.001
Negative life event in last 12 months	1218 (40.1)	49 (57.0)	1169 (39.6)	2.03	1.32; 3.13	0.001
Impaired social role functioning (< 100)	983 (32.4)	59 (68.6)	924 (31.3)	4.93	3.09; 7.85	< 0.001
Impaired physical role functioning (< 100)	624 (20.6)	40 (46.5)	584 (19.8)	3.68	2.37; 5.71	< 0.001
Impaired emotional role functioning (< 100)	309 (10.2)	37 (43.0)	272 (9.2)	7.67	4.90; 12.03	< 0.001

^a^primary basic vocational /lower secondary versus Higher secondary/ Higher professional/ university

## Discussion

### Main findings and interpretation

In this large representative general population sample of family caregivers caring for people with a variety of illnesses and disabilities, no significant difference was found in the rate of suicidal thoughts between caregivers and non-caregivers. In caregivers, the risk factors for suicidal thoughts were limited resources (no paid employment, living without a partner, and lower levels of social support) and poor health. These risk factors were also found in non-caregivers. There was no significant association between suicidal thoughts and specific caregiving characteristics, such as type of caregiving.

Our finding that the rate of suicidal thoughts between caregivers and non-caregivers did not differ is in line with a previous, relatively small study among a family caregivers of people with dementia in Japan [6], but contradicts with two previous studies with large representative samples, which found that caregivers had higher rates of suicidal thoughts than non-caregivers [12, 14]. Differences in study design and population may be the simplest explanation for this discrepancy. For example, it could be that the study of Stansfeld and colleagues included a more vulnerable population of caregivers, as they were exposed to high levels of stressors such as debt problems, domestic violence, and dealing with alcohol problems, which were associated with increased risk of common mental disorders. The caregivers participating in the study of O’Dwyer and colleagues were all middle-aged women. Caregiver literature has consistently shown that female caregivers are more burdened than male caregivers (e.g. [26, 27]. This may explain the relatively high rate of suicidal thoughts (7.1%) in this caregiver sample and the significant difference with non-caregivers in that study. In addition to differences in sample characteristics, more complex social, cultural and political factors that might influence the rate of suicidal thoughts and behaviours in caregivers cannot be ignored. For example, in The Netherlands, comprehensive care and support services are available, and compared with Australia, the country has fewer remote and isolated areas. This could be a reason why the rate of suicidal thoughts between caregivers and non-caregivers did not differ significantly in this study. Comparative studies across countries might help to disentangle the role of social, cultural, and political factors.

The risk factors for suicidal thoughts in caregivers identified in the current study are broadly consistent with previous research. Poor physical health, poor mental health, and a lack of social support have all been previously identified as risk factors for suicidal thoughts in family caregivers [5, 7, 8, 11, 12, 14, 16] and should be considered clear targets for prevention and intervention in policy and practice. The evidence for employment status as a risk factor has, however, been mixed in previous studies. In a study of people caring for children with HIV in Malawi and South Africa, Skeen et al. (2014) found that caregivers living in a house where no-one was employed were more likely to report suicidal thoughts than those living in a house where someone was employed [9]. Similarly, in a study of people caring for family members with cancer in Korea, Park et al. (2013) found that becoming unemployed while caring increased the likelihood of suicidal thoughts [11]. These findings are in line with the results of the current study, where caregivers without paid employment were more than twice as likely to contemplate suicide than caregivers in paid employment. In contrast, O’Dwyer et al. (2016) found no association between employment and suicidal thoughts in people caring for family members with dementia [7], and Rosengard and Folkman (1997) found no association between employment and suicidal thoughts in men caring for partners with HIV [8]. Both studies did, however, find associations between income and suicidal thoughts, with lower incomes and difficulty managing on current income, respectively, associated with greater suicidal thoughts. Although employment and income are closely linked, they are not the same thing. Employment, for example, may offer caregivers an identity, purpose, and source of social support beyond their caring role, while income (at least in some countries) may allow caregivers to purchase additional support. Future studies may need to measure both income and employment in order to gain a more accurate understanding of the way these factors influence suicidal thoughts in caregivers and how these might be addressed in policy and practice.

We did not find a significant association between reason for care (physical disorder, mental disorder etc.) and suicidal thoughts, nor between type of caregiving (domestic, personal, emotional etc.) and suicidal thoughts. The association between suicidal thoughts and caring for someone with a mental disorder did, however, approach significance (*p* = 0.07). In a study of caregivers in Taiwan, Huang and colleagues (2018) found that people caring for a family member with a mental disorder reported significantly higher rates of suicidal thoughts than those caring for a family member with a physical disorder [16]. Also other previous studies indicated that rates of suicidal thoughts might vary according to the condition requiring care. The lack of significant association in the current study therefore may be due to a lack of statistical power.

The current study also found no significant association between suicidal thoughts and other caregiving-related variables such as the relationship with the care recipient, caring for more than one care recipient, and the duration and amount of care provision. This is consistent with previous Dutch research which found no association between suicidal thoughts and variables such as living with the care recipient, length of time spent caring, and ability to leave the care recipient alone [5]. In studies outside the Netherlands, however, some caregiving-related variables have been associated with suicidal thoughts. O’Dwyer and colleagues (2014), for example, found a significant association between (dis) satisfaction with the caring role and suicidal thoughts [12], and Huang and colleagues (2018) found a significant association between (lack of) support from co-caregivers and suicidal thoughts [16]. Huang and colleagues also found an association between the age of the care recipient and suicidal thoughts, with younger ages associated with increased likelihood of suicidal thoughts. Previous studies have been inconclusive about the relationship with the care recipient. Abbott and colleagues (2014), for example, found that in people caring for a family member with cancer, spousal caregivers were significantly more likely to contemplate suicide than other family caregivers [15], but O’Dwyer and colleagues (2016) found no association between the caregiver-care recipient relationship and suicidal thoughts in people caring for family members with dementia [7]. Although differences in the variables measured in each study are the most parsimonious explanation for these discrepancies, the impact of different health and social care systems, as well as cultural values and political context, across countries cannot be discounted [5, 9, 17, 28, 29]. Future studies may need to take a more consistent approach in measuring caregiving characteristics to allow for better comparisons across studies and across different illnesses and disabilities. Studies that compare rates across countries may also help to disentangle the role of cultural and socio-political factors.

### Strengths and limitations

The current study has several strengths that make it a valuable contribution to the caregiving literature. We used a large, representative sample from the general population and compared the rate of suicidal thoughts and risk factors between caregivers and non-caregivers. Only two previous studies have done this before [12, 14]. We also looked at suicidal thoughts over time. In contrast with most other studies, our sample included people providing care for people with physical problems, mental problems, ageing, and dementia. A wide range of potential risk factors, including caregiving and non-caregiving characteristics, were examined.

Despite these strengths, the current study also has some limitations. Although the overall sample was large and the period of observation was 4 years, the number of caregivers reporting suicidal thoughts was small. This limited our ability to conduct multivariate analysis of risk factors. To limit possible confounding effects as much as possible, however, we adjusted for age and gender in the regression analysis. It is still possible, however, that the power was too small to detect a relationship between certain factors (such as providing care for a person with a mental disorder) and suicidal thoughts. Future research would benefit from samples that facilitate multivariate analysis, although this is complex when studying relatively rare events such as suicidal thoughts.

Furthermore, because of the relatively small number of caregivers reporting suicidal thoughts, it was not considered appropriate to examine possible recurrence of suicidal thoughts or report the rate of suicide attempts in this population. Despite this, understanding how suicidal thoughts arises and persists across the caregiving trajectory, as well as how suicidal thoughts are related to suicide attempts in this population, remains important for the development of tailored prevention and intervention strategies. Previous research has shown that suicidal thoughts can occur while caring at home, following institutionalization, and after bereavement [5, 7] and that up to 20% of suicidal caregivers say they will attempt suicide in the future [7], but more research is required to understand the events that precipitate suicidal thoughts and prompt suicide attempts at different stages of the caregiving journey.

The presence of suicidal thoughts was based on retrospective self-reports over the last 12 months (at T1) and the previous 3 years (at T2). Due to this relatively long time span, recall bias may have occurred and resulted in underreporting of suicidal thoughts. However, recall bias is not likely to differ between caregivers and non-caregivers in this study and therefore it is unlikely that this has influenced the comparison of the rate and risk factors of suicidal thoughts between caregivers and non-caregivers.

Lastly, respondents were defined as informal caregivers if they provided unpaid care in the twelve months preceding the second wave (T1), but no data on their caring status 3 years later was available. It might be possible that some people were no longer providing informal care at the second wave and this may have influenced the presence or absence of suicidal thoughts.

## Conclusion

There is a small but rapidly growing body of research on suicidal thoughts in family caregivers and the current study makes a substantial contribution. The absence of any significant difference in the rate of suicidal thoughts between caregivers and non-caregivers stands in contrast to previous findings and highlights the need for more nuanced research to understand this complex phenomenon. Research that can identify which carers are at greater risk of suicidal thoughts, and when, will be key to developing effective intervention and prevention strategies. In the meantime, the current findings suggest that at the least, caregiving is not a protective factor, with caregivers no less likely to consider suicide than non-caregivers. Risk factors of suicidal thoughts in informal caregivers were largely similar to the risk factors in non-caregivers. The current findings suggest that caregivers with limited resources and poor health might benefit from prevention and intervention efforts. Finally, quantitative studies do not allow consideration of the broader social context in which both caring and living with a disability take place [29]. Qualitative methods may prove valuable in exploring these issues in the future.

## Data Availability

The data on which this manuscript is based are not publicly available. However, data from NEMESIS-2 are available upon request. The Dutch ministry of health financed the data and the agreement is that these data can be used freely under certain restrictions and always under supervision of the Principal Investigator (PI) of the study. Thus, some access restrictions do apply to the data. The PI of the study is second author of this paper and can at all times be contacted to request data. At any time, researchers can contact the PI of NEMESIS-2 and submit a research plan, describing its background, research questions, variables to be used in the analyses, and an outline of the analyses. If a request for data sharing is approved, a written agreement will be signed stating that the data will only be used for addressing the agreed research questions described and not for other purposes.

## Abbreviations

CIDI: Composite International Diagnostic Interview
HIV: Human immunodeficiency virus
NEMESIS: Netherlands Mental Health Survey and Incidence Study
SF-36: Short Form-36
SPSS: Statistical Package for the Social Sciences
UK: United Kingdom
USA: United States of America
